# Temperature Influence on Additive Manufactured Carbon Fiber Reinforced Polymer Composites

**DOI:** 10.3390/ma14216413

**Published:** 2021-10-26

**Authors:** Isyna Izzal Muna, Magdalena Mieloszyk

**Affiliations:** Institute of Fluid Flow Machinery, Polish Academy of Sciences, Fiszera 14, 80-231 Gdansk, Poland; imuna@imp.gda.pl

**Keywords:** temperature influence, additive manufacturing, composite, fused deposition modelling, carbon, finite element method, fibre Bragg grating

## Abstract

The popular applications of Additive Manufactured (AM) polymer materials in engineering, medical, and industrial fields have been widely recognized due to their high-speed production despite their complex design shapes. Fused Deposition Modeling (FDM) is the technique that has become the most renowned AM process due to its simplicity and because it is the cheapest method. The main objective of this research is to perform a numerical simulation of the thermo-mechanical behaviour of AM polymer with continuous carbon fibre reinforcement exposed to elevated temperatures. The influence of global thermal loads on AM material was focused on mechanical property changes at the microscale (level of fiber–matrix interaction). The mechanical response (strain/stress distribution) of the AM material on the temperature loading was modelled using the finite element method (FEM). The coupled thermal-displacement analysis was used during the numerical calculations. The strain in the sample due to its exposition on elevated temperature was measured using fibre Bragg grating (FBG) sensors. The numerical results were compared with the experimental results achieved for the sample exposure to the same thermal conditions showing good agreement. A strong influence of the temperature on the matrix structure and the condition of bondings between fibres and matrix was observed.

## 1. Introduction

The emerging evolution of additive manufacturing (AM), also known as 3D printing, has been immensely fascinating since the birth of its first conception in 1981 using the stereolithography process, which then became patent and commercial in 1986 [1,2,3]. There are numerous applications of AM elements in various fields, e.g., aerospace [4], medical [5], dentistry [6], consumer goods [7], renewable and nuclear energy [8,9], as well as energy storage devices such as battery [10,11,12] and fuel cell [13,14,15,16]. The AM term has been prevalent for a wide range of techniques used to fabricate a variety of 3D shapes by depositing an additional material layer upon layer in contrast to the subtractive method. With rapid prototyping of AM, it provides a possibility to construct the complex geometrical elements with some benefits such as more improved speed and lower cost integration process which enables testing of new design concepts [17]. Besides, other advantages of the AM process include the potential of material waste reduction and sustainable energy consumption [18].

In recent years, the Fused Deposition Fabrication (FDM) process has been the most known technique among AM methods due to its simplicity and lowest cost of production. This process is included in material extrusion since it creates objects by melting filament and extruding it through a small rastering nozzle that moves in three dimensions onto the building platform. The deposited semi-molten material then cools rapidly, creates solidification, and bonds with the adjacent rasters, whereas a change of phase occurs simultaneously. In the next step, the platform is lowered, and the next layer is extruded onto the top of the previous layer. The process is repeated until the final part is obtained [19]. The quality of the printed parts depends on various underlying physical phenomena during printing, while the part integrity and properties depend highly on bonding phenomena and bond quality. Once the printing process is completed, the final FDM component will form vertically stacked layers (interlayer) of a laminate composite structure which are bonded together. The bond formation between two layers includes surface contacting, neck growth and molecular diffusion [20].

The FDM method is commonly used for the manufacture of polymer-based materials. Nevertheless, the properties of the pure (neat) polymers components printed using FDM are still lacking mechanical strength and functionality which make them being fabricated for prototyping samples [21]. With considerable attention to the advancement of composite materials, improving the properties of FDM printed parts by developing composite systems as the reinforcement of different fillers to the base polymer is essential. In composite materials, two constituents are required: Matrix (thermoplastic polymers) and reinforcement agent. Recently, there has been a flourishing of experimental works in the fabrication of various 3D printed composites using FDM with carbon-based reinforcements have been performed to enhance the mechanical properties compared to the non-reinforced (neat) polymer printed parts [20,22,23,24,25,26,27,28,29,30].

Carbon fiber reinforced polymer (CFRP) is highly applied in the engineering field among any other type of reinforcement material, owing to its improved tensile strength, lightweight, greater stiffness, and low thermal expansion [26,31]. In continuous fiber reinforced composites, the poor adhesion between matrix and fiber interface deteriorates mechanical properties. Tian et al. [28] proposed continuous carbon fiber reinforced thermoplastic (CCFRTP) materials by employing continuous carbon fiber and polylactic acid (PLA) filament which was fed into the FDM 3D printing process simultaneously. During the forming process, the influence of process parameters, such as temperature and pressure, is important to study the interfaces and performance of printed composites. Interface and carbon fiber content are technical factors for the composites specifications. It was found that flexural strength of 335 MPa and modulus of 30 GPa were obtained when the fiber content reached 27% [28]. In similar work, Matsuzaki et al. [26] developed a method for 3D printing of CCFRTP based on FDM using continuous carbon fiber tows and jute twisted yarns as reinforcements in PLA as the matrix which enables direct 3D fabrication without the use of moulds. It was shown that the tensile strength and modulus of CCFRTP composite printed parts are higher than the values shown by conventional composite printing techniques. It is also reported that the mechanical properties of these parts can be further improved by increasing the volume fraction of continuous fibers [26].

Recently, Zhou et al. [32] conducted an experiment on the longitudinal thermal expansion deformation and the mechanical properties of CFRP tendons with 8 mm diameter in the steady state and transient state. It was obtained that the longitudinal deformation of CFRP tendons is negative at high temperature due to resin softening, and the transient state test results are slightly higher than at the steady state. Furthermore, a new constitutive model of CFRP tendons at elevated temperature was also proposed based on the analysis of thermophysical properties. The model result was presented with the numerical fitting technique which is well-agreed with the experimental results. An experimental investigation of the mechanical response and failure process of the unidirectional CFRP composites at elevated temperature was also performed by Jia et al. [33] using static and dynamic three-point bending tests. It was revealed that although CFRP possesses relatively poor performance at a higher temperature (100 ${}^{{}^{\circ}}$C), but it provides enhanced flexural strength, maximum deflection, and energy absorption at lower temperatures (60 ${}^{{}^{\circ}}$C, 100 ${}^{{}^{\circ}}$C). In addition, to understand the underlying mechanisms responsible for these temperature dependent mechanical behaviors, analytical modeling is applied.

A numerical investigation of thermo-elastic behaviour on CFRP at elevated temperatures has been studied by Ejeh et al. [34] using heat transfer analysis coupled with material layer-wise arrangement technique. The results revealed that thermal stresses are intense along fiber-direction of the composite laminates. Moreover, the CFRP composite material also offers good resistance to thermal stress, even at elevated temperatures up to 1070 ${}^{{}^{\circ}}$C. Luders et al. [35] presented an experimental and numerical multiscale approach for investigating thermally cycled FRP which contributes to the understanding of the damage mechanisms and increase the predictability of the fatigue damage at thermal loading conditions. The cycled thermal loading of FRP is investigated at the microscale (level of fibre–matrix-interaction) and macroscale (level of the multidirectional laminate). It was suggested to use micromechanical fatigue modelling based on RVE approach to explain the differing experimentally observed crack propagation in FRP obtained for mechanical and thermal fatigue loading.

So far, some research on the mechanical behaviour of CFRP has been investigated during the printing process with the FDM method and not subjected to the 3D printed components itself. In addition to that, there is still limited exploration on the temperature effect on the mechanical behaviour of CFRP materials at elevated temperatures up to the heat deflection temperature of PLA as of 55 ${}^{{}^{\circ}}$C. The novelty of the current research is the investigation of the temperature effect on the mechanical behaviour of 3D printed CFRP composite. The goal of this paper is to model the 3D printed materials with carbon fiber reinforcement using Abaqus, the Finite Element Analysis software package. Moreover, this paper expanded the investigation in numerical methods in order to analyse the thermo-mechanical behaviour of the sample model which is exposed to elevating global thermal loading. The resulted simulation was validated using experimental work performed at an environmental chamber using fibre Bragg grating (FBG) embedded into the CFRP material structure.

## 2. Materials and Methods

### 2.1. Numerical Modelling

#### 2.1.1. Model Description

The numerical investigation was carried out to simulate the behaviour of polymeric composites in relation to the effect of temperature on the samples. The 3D printed CFRP was modelled as stacked solid elements at laminate level, and considered as a homogeneous equivalent material from a macromechanics standpoint. The laminate consists of six-ply unidirectional (UD) lamina with stacking sequence (LSS) at 0 degree and the constitutive material behaviour of laminate was considered as a single orthotropic material. The array of carbon fibers in polymer matrix assumed in square arrangement as it will have symmetry planes parallel and perpendicular to the fibers. This type of material has three mutually perpendicular planes of symmetry and possesses nine independent elastic constants [36]. To describe the deformation and mechanical behaviour of material macro-mechanical modelling was used.

To obtain the numerical results for the CFRP, a finite element method (FEM) model was used. The composite model geometry was created using the computer aided design tool Abaqus software as a deformable homogenous solid. The cross-sectional area of model geometry is 9.2 mm × 9.5 mm with the thickness of 2 mm. The specimen model was discretized into hexahedral mesh of eight-node trilinear heat transfer brick with C3D8T element type. The FEM model consisted of 4416 finite elements, 19,266 nodes, and 249,750 degrees of freedom. The simulated load scenario consisted of two stages such as initial step and loading step, corresponding to temperatures ranging from 10 ${}^{{}^{\circ}}$C to 40 ${}^{{}^{\circ}}$C with a step of 5 ${}^{{}^{\circ}}$C. It was assumed that the relative humidity inside the chamber is stable at 20%. The numerical modelling of CFRP composite using the coupled temperature-displacement approach will be investigated within thermo-mechanical strain analysis during the elevated heating.

#### 2.1.2. Material Properties

The constitutive material behavior of each layer of the printed part is orthotropic and similar to lamina behavior. Mechanical behavior of printed parts can be characterized using classical laminate theory, and these studies have focused on only one kind of build strategy, namely flat build orientation [37]. In orthotropic materials, symmetry properties with respect to certain planes are exhibited. This means that when the direction normal (or perpendicular) to the plane of symmetry is reversed, the elastic constants do not change.

Assumed material properties are listed in Table 1.

The elastic properties for a unidirectional composite laminate can be calculated using the rule of mixture (ROM) formula as being orthotropic material. A UD lamina was sufficiently approximated as being transversely isotropic where it suffices to use $E_{33}$ = $E_{22}$, and $G_{23}$ = $E_{33}$/2(1 + $\nu_{23}$) in the equations for an orthotropic material. The elastic properties (Table 2) of a unidirectional lamina were computed using experimental data of whole laminate from the experimental analyses performed at the Kaunas University of Technology who developed the FDM method and manufactured the sample for the experimental validation of the proposed numerical model [38].

For a transversely isotropic material such as a UD reinforced fibrous composite, the determination of thermal conductivity in the composite material can be predicted by the ROM type expression [39]. The parallel thermal conductivity in the axial direction (longitudinal to fiber) and the series conductivity in the transverse direction (perpendicular to fiber) are calculated using the formula in Equation (1) and Equation (2), respectively:

(1) $$\kappa_{11}=V_{f}\kappa_{f}+V_{m}\kappa_{m}$$

(2) $$\kappa_{22}=\frac{\kappa_{f}\kappa_{m}}{V_{m}\kappa_{m}+V_{f}\kappa_{f}}$$

While for the heat capacity of composite is calculated using the following relationship:

(3) $$C_{v}=\frac{\left(V_{f}\rho_{f}C_{f}+V_{m}\rho_{m}C_{m}\right)}{\rho (V_{f}+V_{m})}$$

The indexes *f* and *m* means fibre and matrix, respectively. *V* is the volume and ρ means density. The volume fraction has to fulfil the relationship $V_{f}+V_{m}=1$.

The following are the basic assumptions considered in the thermal case based on Schapery formulation [40]:

- fibre and matrix are assumed to be isotropic and linear elastic.
- The strains in the longitudinal direction are the same in the matrix and in the fibres (Voigt assumption)
- The stresses in the transverse direction are constant (Reuss assumption)
- The homogenised macroscopic stresses in the lamina are zero, i.e., the assumption δL = δT = 0 is used.

Thermal properties of the CFRP composite are collected in Table 3.

The ROM equations for predicting thermal properties of CFRP result in lower thermal transverse conductivity compared to in-plane conductivity. As it has been discussed by Fan et al. [41], it should be noted that the transverse thermal conductivity of carbon fiber increases with a decrease in the volume fraction of the fiber (*V*${}_{f}$), while for in-plane thermal conductivity it increases with increasing *V*${}_{f}$, which corresponds well to the thermal conductivity of Fiber Reinforced Plastics (FRPs) [41].

#### 2.1.3. Boundary Problem Formulation

**Figure 1:**
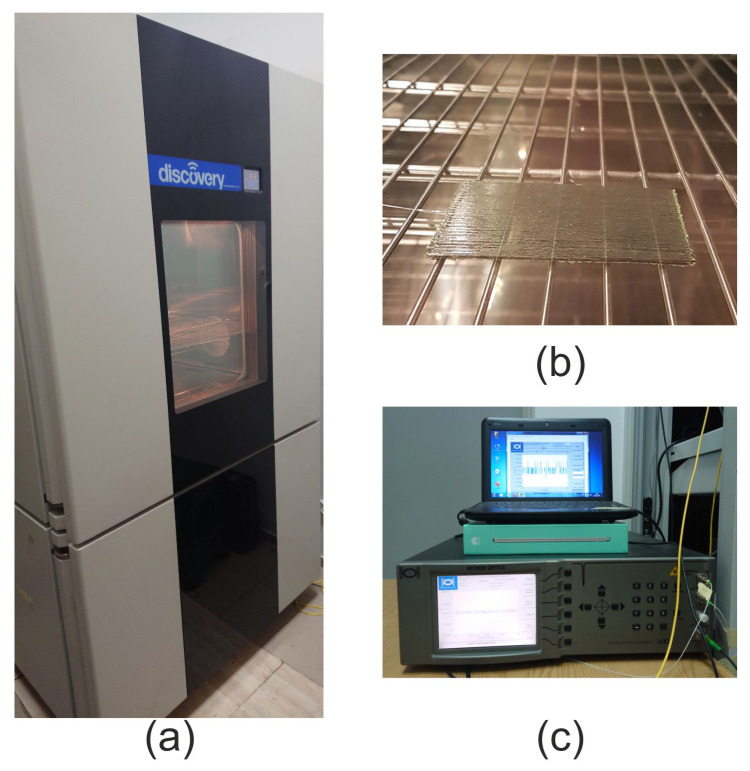
Measurement set-up: (**a**) Environmental chamber, (**b**) sample placed on a shelf inside the chamber, (**c**) interrogator with the computer.

In setting up the boundary conditions, the model sample is on a shelf inside the chamber, see Figure 1b with displacement boundary U3 = 0 at the bottom of the sample (rectangular surface) laying on a shelf, U2 = 0 at one side, and U1 = 0 at another side. The initial temperature of sample is set as 20 ${}^{{}^{\circ}}$C at the entire sample location within the predefined field feature of load module in Abaqus, whereas the temperature inside the chamber was assumed to be in a range from 10 to 40 ${}^{{}^{\circ}}$C with 5 ${}^{{}^{\circ}}$C increment.

The sample will possess two types of heat transfer schemes. First is thermal radiation from the chamber to the sample surfaces, followed by heat conduction between the material layers. The heat sample module in Abaqus was used simultaneously with the heat transfer physics. The initial temperature is set to 20 ${}^{{}^{\circ}}$C at all nodes and loading temperature is applied at a range from 10 ${}^{{}^{\circ}}$C to 40 ${}^{{}^{\circ}}$C with 5 ${}^{{}^{\circ}}$C increment. The boundary conditions assigned to the sample model are determined by how it interacts with the external surroundings to accurately represent the physical phenomena of the experimental set-up which may insulate the edges. The heat was applied at the top surface of the laminate and is distributed from the top layer (layer 1) towards the bottom (layer 6) with the Stefan–Boltzmann constant = 5.67 × 10${}^{-8}$ Wm${}^{-2}$K${}^{-4}$ and emissivity ϵ = 0.96 [42]. In addition, the sample orientation was positioned correspondingly with the global axis, which identifies the different faces of the laminate.

It is worth to mention that in this modeling procedure the convection heat transfer was not considered. In environmental chamber temperature is controlled by an electric heater, where the mechanism of heat exchange is propagated through electromagnetic waves. Thus there exist no heat flow that can be brought to the wall of sample or taken away from it.

With the initial value problem now in a suitable format, the equations were discretized to yield the finite element equations. The final step is to adopt an isoparametric approach and to introduce the appropriate quadrature scheme, which will give us the equations to be solved numerically [43].

#### 2.1.4. Non-Linear Transient Heat Transfer Formulation

Based on the law of conservation of energy, the transient heat transfer problems in a time-continuous spatial domain which physically interpreted as a heat flow equilibrium statement [44]. The non-linearity arises with the presence of radiation boundary conditions and the temperature dependence of the material properties, considering that there is no contact between the boundary of the domain (composite structures) with some fluids.

The Finite Element Method is based on the discretization (approximation) of the weak (variational) form of the partial differential equations which is derived from the classical form. The strong-form equation governing transient heat transfer problems in the system Ω can be written as

(4) $$\rho c\left(T\right)\frac{\partial T(x,t)}{\partial t}=\frac{\partial }{\partial x_{i}}\left(k_{ij}\left(T\right)\frac{\partial T(x,t)}{\partial x_{j}}\right)+Q\left(x,t\right)$$

where ρ is the density of material; *c(T)* is heat capacity which is dependent on temperature field at spatial point *x*∈Ω that varies with time *t*; *x* is the spatial coordinates in an *n*-dimensional problem where *x* = ($x_{1}$, $x_{2}$, …, $x_{n}$); $k_{ij}$(*T*) is the thermal conductivity in the direction of the *i*-th component of the spatial coordinates and *j*-th component of the temperature gradient. For the conductivity of orthotropic material $k_{ij}$(T) = 0 for *i*≠*j*; Q(x,t) denotes the volumetric heat flow per unit time; and $t_{0}$ is the initial time.

The Galerkin weak-form finite element method can solve the field problem of transient heat transfer using the matrix form of the discretized transient heat transfer problem and the related initial and boundary conditions [45]. To approximate the governing equations of transient heat transfer, the problem domain is divided into a finite number of subdomains or elements, generating a finite element mesh in one, two, and three dimensions that correspond to the problem domain. Separate functions with common values at element system nodes interpolate the field variables [44]. Individual finite element equations are combined to form a large system of equations from which the unknown field variable values (nodal temperatures) are determined as solutions.

### 2.2. Experimental Method

#### 2.2.1. Materials and Sample

**Figure 2:**
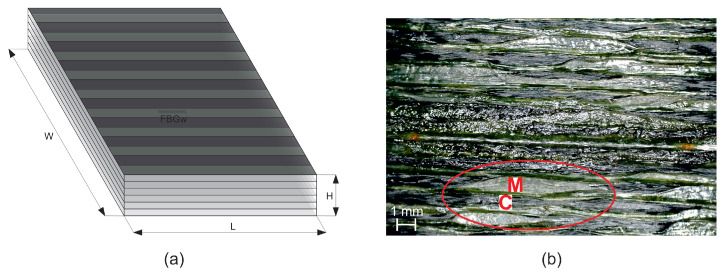
Sample: (**a**) Scheme with FBG sensor locations, (**b**) surface photograph; C—carbon fibre bundle, M—polymeric matrix.

The carbon fiber used as reinforcement phase was T300B-1000 (1000 fiber filaments in an untwisted tow) from Toray (Paris, France) with tensile strength is of 3530 MPa, Young’s modulus of 230 GPa and density 1.76 g/cm [46]. For the matrix agent, PLA 3D850 filaments from Natureworks (Blair, Nebraska, USA) were selected as received for the optimization of the 3D printing process and to establish a baseline with tensile strength is of 51 MPa, Young’s modulus of 2315 MPa and density 1.24 g/cm [46]. The continuous fiber reinforced composite sample was manufactured at the Kaunas University of Technology using 3D printer MeCreator 2 by Geeetech [38]. The printing machine parameters are as follow: Nozzle diameter is 1.5 mm, printing speed is 3 mm/s, bed temperature is 70 ${}^{{}^{\circ}}$C, extruder temperature is 200 ${}^{{}^{\circ}}$C, extrusion multiplier is 0.6, and extrusion width is 1.2 mm. The dimension of unidirectional composite laminate is 9.2 mm × 9.5 mm × 2 mm with six plies and stacking sequence [0, 0, 0, 0, 0, 0]. The sample scheme and photography are presented in Figure 2. Due to the small thickness of the carbon fibre bundles, there was a problem during AM process that results in the uneven distribution of polymeric matrix and carbon fibre in the sample volume. Additionally, the fibres are partially rotated. It is well visible in the sample surface photograph in the part marked by the red ellipse. In the middle of the sample (between the 3rd and the 4th layers) an FBG sensor (Micron Optics, Atlanta, GA, USA) was embedded in order to examine the strain resulted during the thermal testing.

**Figure 3:**
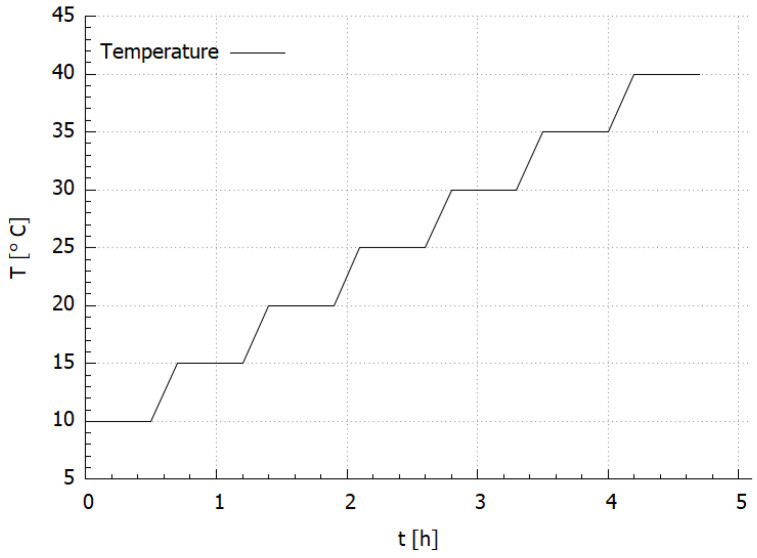
Program of the test.

For thermal testing, the sample was put inside the environmental chamber (MyDiscovery DM600C (Angelantoni Test Technologies Srl, Massa Martana, Italy)) where the temperature was set in a range from 10 to 40 ${}^{{}^{\circ}}$C with 5 ${}^{{}^{\circ}}$C, increment under a stable value of the relative humidity (RH) level at 20% as it is presented in Figure 3. For the FBG sensors measurement interrogator si425-500 (Micron Optics, Atlanta, GA, USA) with a measurement frequency equal to 1 Hz was used. The temperature close to the sample was measured using an FBG temperature probe (Micron Optics, Atlanta, GA, USA). The measurement set-up is presented in Figure 1.

#### 2.2.2. Measurement Method

In this work, the selected measurement method to determine the thermal expansion was using FBG sensor. This sensor was integrated with manufactured sample of PLA-CFRP composite via FDM process. FBG sensor was selected owing to their advantages of small size and low weight, lack of calibration requirements, and high chemical resistance [47]. Moroever, the embedded FBG sensor is part of structural health monitoring (SHM) systems, are useful in monitoring the behavior of elements during their manufacturing and subsequent exploitation (tests) processes. They allow gaining useful information for future design (modifications) [47].

Previously, a similar method was employed by Kousiatza et al. [19]. They analyzed the integration of FBG sensors with structures manufactured using the FDM technology for acrylonitrile butadiene styrene (ABS). Simultaneous monitoring of strain and temperature profiles of an embedded optical sensor was performed. Strain and temperature were measured using an FBG sensor during the embedding process. Recently, Mieloszyk et al. [47] investigated an embedded FBG sensor in polymeric structure printed using Multi-jet printing (MJP). It was reported that the MJP is appropriate for constructing elements with embedded FBG sensors. Moreover, MJP is also able to determine the relationship between strain and temperature as well as between strain and relative humidity.

## 3. Results

Numerical simulations using a non-linear solver were conducted and results are presented in this section in comparison with the experimental result.

### 3.1. Experimental Results

The total strain $\varepsilon_{c}$ was calculated using the following equation:

(5) $$\varepsilon_{c}\left(T\right)=\frac{\lambda_{m}\left(T\right)-\lambda_{b}\left(T\right)}{\lambda_{b}\left(T\right)}$$

where $\lambda_{m}$ and $\lambda_{b}$ are measured and base Bragg wavelengths, respectively. As base conditions, RH equal to 20% and room temperature (20 ${}^{{}^{\circ}}$C) were assumed.

Strain values for the CFRP in the FBG sensor locations were determined using the following relationship:

(6) $$\varepsilon \left(T\right)=\varepsilon_{c}\left(T\right)-\varepsilon_{f}\left(T\right)$$

where indexes *S* and *f* are related to the FBG sensor embedded into the sample and free, respectively. The relationship between the FBG sensor and temperature was determined experimentally.

**Figure 4:**
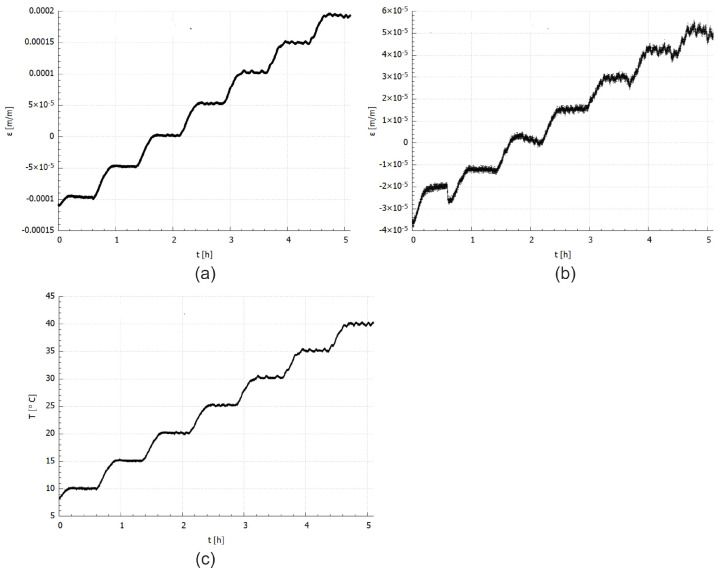
Experimental results: (**a**) CFRP total strain, (**b**) CFRP material strain, (**c**) temperature.

Temperature and strain values determined from FBG sensors measurements are presented in Figure 4. It is well visible that the strain inside the CFRP material increase with temperature (Figure 4c), even when the influence of the temperature on the fibre optic material and sensor is removed. The shapes of all curves (temperature and strain) are similar.

The CFRP material strain was then used for the determination of the relationship between strain and temperature and transform into point values using the following formula:

(7) $$\varepsilon_{T}=\frac{1}{n}\sum_{i=1}^{n}\varepsilon_{n}\left(T_{i}\right)\;for\;i=1,\cdots ,7;\;n=n_{1},\cdots ,n_{n};$$

where temperature level $T_{i}$ is related to averaged temperature value from *n* points for stable temperature conditions lasting 300 s. The calculation error for temperature was 0.29 ${}^{{}^{\circ}}$C, whereas that for the strain was 3.33 × 10${}^{6}$ m/m. The measurement accuracy of the interrogator is equal to 1.0 × 10${}^{6}$ m/m, so the differences are neglected.

**Figure 5:**
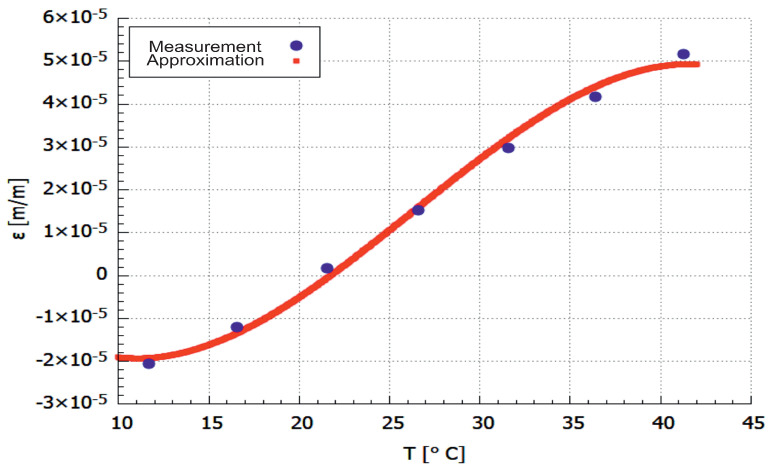
Strain in CFRP material determined experimentally.

The points were approximated using the 3rd order polynomial

(8) $$\varepsilon \left(T\right)=C_{1}T^{3}+C_{2}T^{2}+C_{3}T+C_{4}$$

where the *T* is temperature, ε is the strain, while $C_{1}$…$C_{4}$ are polynomial constants listed in Table 4. The approximated strain curve is presented in Figure 5.

The average percentage deviation between the points determined experimentally (acc. to Equation (7)) and approximation (Equation (8)) was calculated using the following relationship:

(9) $$\varepsilon_{e}\left(T\right)=\frac{\varepsilon_{E}-\varepsilon_{A}}{\varepsilon_{E}}$$

where indexes *E* and *A* refers to experimental and approximated values of the strain, respectively. The mean value of the error for the sample for all analysed points was equal to 2.7%.

FBG sensor was placed in the middle and embedded parallelly to the main axes of the sample. The obtained strain values in the form of a function (Equation (8)) allow determining the thermal expansion. The relation can be obtained from the first derivation of strain function, thus the coefficient of thermal expansion (CTE) is also obtained.

The atomic or molecule vibration is responsible for a medium position in any material. As the material temperature rises, thermal energy-induced vibration increases in amplitude and interatomic or intermolecular distances increase, i.e., the body expands [48]. The CTE coefficient function is presented for whole range of the measured temperature, also with the part that due to matrix properties the CTE parameter value decrease with the temperature increasing.

### 3.2. Model Validation

In order to determine the accuracy of the numerical model and to achieve the simulation reliability, convergence analysis and mesh resolution (grid-independent) have been performed. Mesh convergence establishes how many elements are required in a model to ensure that changing the size of the mesh has no effect on the outcomes of a study. With decreasing element size, the system response (stress, deformation) will converge to a repeatable solution. Grid-independent indicates that the calculated results slightly change when the grid becomes finer (denser) or coarser (looser) such that the truncation error can be ignored in numerical simulation. The independence of the grid has a direct impact on the truncation error and even the logic of numerical results [49]. The grid or mesh is said to be grid-independent if the mesh resolution is increased to a specific ratio for each different simulation, then the results tend towards identical. Additional mesh refinement has no effect on the outcomes after convergence. At this point, the model and its output are independent of the mesh. Such an approach can make the best use of computer resources while producing appropriate outcomes.

The good meshing feature can be attained by changing seed (node) and mesh control such that the model will have more elements where stress change is high while fewer elements where stress change is low to avoid the high computational cost and no significant change of mesh size or mesh shape. In general, the number of elements is proportional to the simulation time. For the model used in this work, convergence has been obtained for meshing the sample structure with more than 6156 finite elements and a number of nodes of 7220. The seeds are set at the same distance on every edge with an approximate global size of 0.25. In order to reduce the computational cost while keeping high thermal field resolution, a mapped mesh employing hexahedral 8-node elements is applied.

An attempt to check the convergence has been done by generating 6 varieties of meshing density and picking 9 different nodes over the sample model. The solver was not able to proceed with the simulation for two meshing densities with seed sizes less than 0.25. In the non-linear simulation problem, the extremely finer mesh will create a larger stiffness matrix and due to the derived unbalanced forces applied to the system, it might cause the solution to diverge, whereas for the seed sizes bigger and equal to 0.25 the results seem to give similar thermal field values. This means that the simulation model is well-agreed with grid-independent rule and therefore, the mesh convergence is well-satisfied.

**Figure 6:**
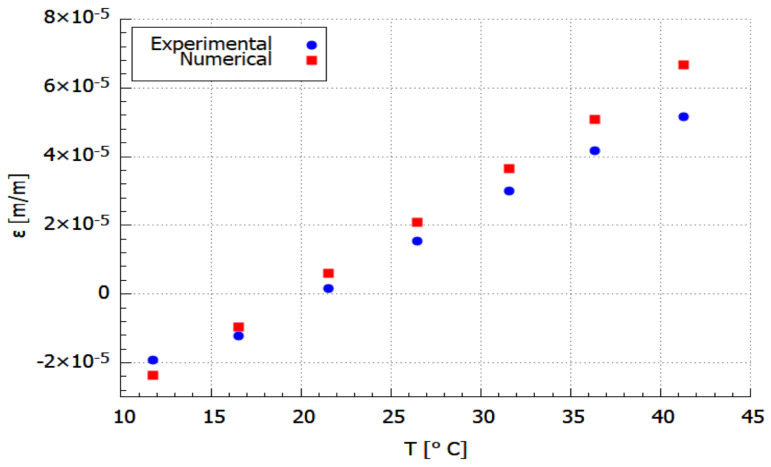
Discrete comparison of experimental and numerical results.

**Figure 7:**
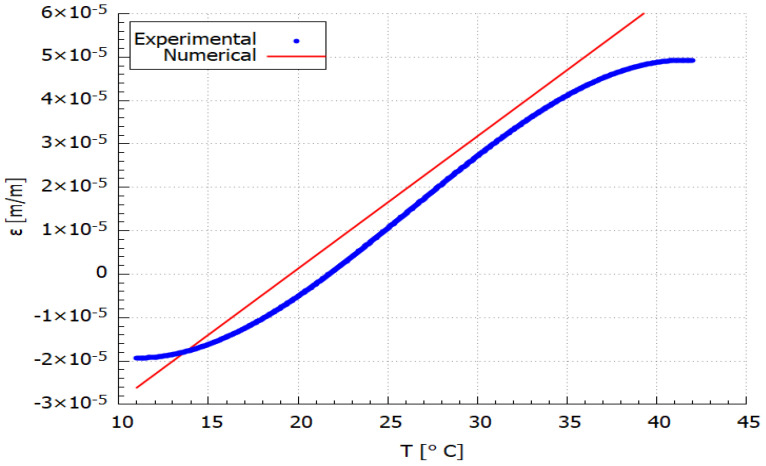
Comparison curves determined experimentally and numerically.

The obtained numerical results of the temperature and strain relationship were compared with the experimental data. The comparative plots are shown in Figure 6 and Figure 7. The results presented in Figure 6 are related to the exact temperature values determined from the temperature measured during the experimental test (Figure 4d) according to the procedure described for Equation (7). While the comparison presented in Figure 7 is linked with the experimental strain approximation that is burdened with 2.7% error (acc. to Equation (9)).

It can be seen from the discrete curve (Figure 6) that a good correlation was obtained between numerical and experimental values. The difference between those values is due to some defects which the specimen possess. Some presented voids and structure irregularity in the sample contributed to the inconsistency during the experimental measurement [20,50,51]. During the FDM printing, the inclusion of reinforcements may enhance the strength of the material system, but this gain is offset by a poor reinforcement/matrix interface, non-uniform reinforcement distribution, and incorrect impregnation. These variables will lead to the development of new voids [20].

Comparing the experimental and numerical values presented in Figure 7 using Equation (9) for the same temperatures it can be observed that the percentage difference between them is ca. 18%, while the average strain difference is equal to 5.7 × 10${}^{-6}$ m/m. The difference between the experimental and numerical curves is related to the heterogeneity of the AM sample structure as it well visible in Figure 2b. Therefore, the numerical model can be applied for the CFRP material thermal strain determination in the analysed range of temperatures.

## 4. Discussion

Several studies have shown that PLA, the polymer matrix employed in this FDM process, has a deflection temperature (HDT) near 55 ${}^{{}^{\circ}}$C [52,53]. HDT is a measure of the resistance of a polymer to deformation (stiffness of material) when subjected to stress loading at elevated temperatures. HDT value in polymer material is determined by the level of crystallinity (morphological structure) and the presence of reinforcing agents such as fillers, plasticizers, etc. In practical applications, the predominance of PLA requires to be reinforced with additives to offset its low impact strength and small HDT. According to scientist, the low HDT is mostly due to the lower glass transition temperature (Tg) of PLA and the slower crystallization rate during manufacturing [54].

PLA is a semi-crystalline type of polymer, which means that PLA has the potential to have more resistance than amorphous. Additionally, the polymer structure of PLA is cross-linked, which means that it may not be easily degraded. It can be seen in Figure 7 that the mechanical properties (strain) increase during the first phase of global heating. This is due to the cross-linking phenomena in polymer material. In the second phase, the excess cross-linking leads to deterioration of the material properties. When subjected to heat, polymeric materials can experience major unrecoverable changes in physical and chemical properties. The chemical processes involved during degradation play a role in the chemical composition of the polymer and also physical parameters such as molecular weight, crystallinity, cross-linking, etc. This may also lead to changes in physical processes like glass transition (Tg), crystallization, and cross-linking [55]. When heating below the decomposition temperature, a thermoplastic material transforms from its glassy state to a rubbery state. Thus, a product comprised of a thermoplastic material will start dripping under such heating conditions. With prolonged heating, once the decomposition reactions start taking place, the Tg of the inherent polymer may alter due to molecular-level structural changes.

Generally, the Tg of PLA lies between 50 ${}^{{}^{\circ}}$C and 70 ${}^{{}^{\circ}}$C [54]. The primary cause of different Tg values within this interval is the polymer morphological feature (structure). Tg is the temperature at which the crystalline or semi-crystalline part of a polymer melts and transitions from an ordered to an amorphous structure. Glass transition is a kinetic rather than a thermodynamic phenomenon.

From the strain-temperature curve in Figure 5, it can be seen that at temperatures around 42 ${}^{{}^{\circ}}$C the sample is no longer possessing an increased strain and is distorted which means that the HDT is situated around that temperature. It is assumed that PLA contributed significantly to the relatively low HDT value as of 42 ${}^{{}^{\circ}}$C with respect to the other references (55 ${}^{{}^{\circ}}$C). As it has been stated previously, Tg has a proportional influence on the HDT value as it depends on the faster or slower crystallization rate.

**Figure 8:**
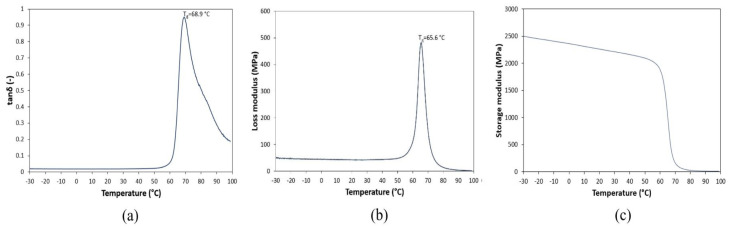
Curves obtained from DMA testing: (**a**) Loss factor (tan δ), (**b**) loss modulus, (**c**) storage modulus.

With regard to analysing how temperature affects PLA and in order to characterize viscoelastic properties of PLA, dynamic mechanical analysis (DMA) testing has been performed. DMA is a method in which a small sinusoidal deformation is given to a sample in a cyclic manner. The instrument used for DMA testing is Q800 DMA-TA with a single cantilever mode and the temperature range was set from −30 ${}^{{}^{\circ}}$C to 100 ${}^{{}^{\circ}}$C. The heating rate was 4 ${}^{{}^{\circ}}$C/min with an amplitude of 20 μm and a frequency of 1 Hz. When dealing with DMA, the heating rate is an important experimental condition, and modest heating rates are necessary to prevent thermal diversification and excessive thermal lag between the sample and the thermocouple [54]. The DMA results are plotted and shown in Figure 8.

The ideal viscoelastic behavior of a thermoplastic elastomer including PLA is divided into glassy region, glass transition region, and rubbery region. Loss factor or damping (tan δ) is defined as the ratio between the loss (viscous) modulus and the storage (elastic) modulus. Loss modulus is related to the flowing properties whereas the storage modulus is connected with the rigidity of the sample. When working with PLA, the glassy area denotes a room temperature state. As a result of the DMA investigation, the storage modulus may be determined under normal operating circumstances. It can be observed from the curves above that glassy region are conveyed by slow descent of storage modulus with an increasing temperature and a rather low and steady tan δ and loss modulus value. In the glassy region, macromolecular chains are frozen in rigid structures and only local motions of bending/stretching are possible. As temperature increases, long-ranged coordinated molecular motion (α-relaxation) is activated and the onset of the glass transition triggers the refolding of polymer chains because their mobility increases [54]. This increasing entropy is the main reason for shrinking [56].

The glass transition region is marked by an area around sharp descent of storage modulus which takes place around 65 ${}^{{}^{\circ}}$C with a sudden increased tan δ and loss modulus similarly at around 65 ${}^{{}^{\circ}}$C. When evaluated on a log scale against a linear temperature scale, the occurrence of a significant drop of storage modulus with tan δ and loss modulus peak is considered as the Tg value of PLA which is at 65 ${}^{{}^{\circ}}$C, approximately. Afterwards, PLA chains have sufficient mobility to develop crystalline regions on the rubbery plateau, which can be shorter or even absent for low-molecular weight PLA [54].

Despite the proportional correlation between heat deflection temperature (HDT) and Tg, it is essential to note that some researchers reported that Tg is very little affected (unperturbed) by addition of reinforcement or filler into PLAs [57,58,59,60]. Consequently, according to the previous statement it is assumed that the Tg value of CFRP will be similar to the Tg of pure PLA obtained from DMA testing which is 65 ${}^{{}^{\circ}}$C. However, the beginning HDT of CFRP has been found to be 42 ${}^{{}^{\circ}}$C which means that its Tg also falls around the aforementioned value. It is well-noticed that PLA reinforced with carbon fiber has lower Tg value and storage modulus than pure PLA. In accordance with several researchers who have performed similar works related to adding filler such as graphene oxide [61] and POSS [62], the reason behind this behaviour is because additional filler has taken space out of PLA chains due to lack of interaction between them. Moreover, additional reinforcement also acts as weak point in the material which caused the formation of shorter chains of PLA. As it has been studied by Cao et al. [63], the extra filler does not act as a fortifier. The refolding of polymer chains caused by enthalpic relaxation may cause the filler to be unevenly distributed across the network, resulting in zones with greater mobility. In this investigation, the contraction of CFRP sample at temperatures above the deflection temperature is a result of enthalpic relaxation at the beginning of the glass transition in the storage modulus curve. The form of the loss modulus accounts for an abrupt halt in mobility growth caused by polymer chain refolding (shrinking) [54]. Furthermore, the effect of plasticizers in PLA blends has also impacted in lower Tg values and reduced storage values in glassy region [64]. Because of the dynamics of events during the glass transition, the tan δ peaks diminish as additional plasticizers are applied, indicating extra-stiffness phenomena caused by shrinking.

Additionally, a pure PLA sample was also measured under the same thermal conditions as the CFRP sample. The PLA sample dimensions were 50 mm × 20 mm × 10 mm and it was manufactured under the same manufacturing conditions as the CFRP sample. Similarly like in the CFRP sample, in the middle of the PLA sample an FBG sensor was embedded.

**Figure 9:**
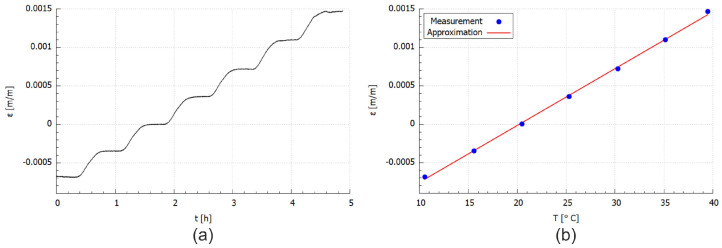
Experimental investigation results for PLA: (**a**) Material strain, (**b**) relationship between the material strain and temperature.

The experimental results for the PLA material are presented in Figure 9. The calculation procedure used for the PLA was the same as it was described above for the CFRP sample. The shape of the strain curve (Figure 9a) is similar to the temperature curve presented in Figure 4. Despite the AM process resulted in an anisotropic, layered structure, the relationship between strain and temperature is linear in the analysed range of temperatures. Therefore, it can be concluded that the observed thermal strain shape determined experimentally for the CFRP sample is related to the influence of the fibre reinforcement on the Tg of PLA and the bondings between the fibre reinforcement and the PLA matrix.

**Figure 10:**
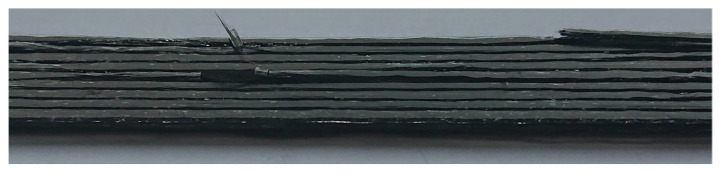
CFRP structure after the tensile test.

Such a conclusion can be supported by the structural degradation process observed for similar CFRP samples (also subjected to the same elevated temperatures) after the tensile test and presented in Figure 10. The only difference between the analysed sample and the tensile test samples was related to their dimensions. They had to meet the requirements of the tensile test standard. It is well visible in Figure 10 that some of the carbon fibre bundles are separated because of the debonding between fibres and matrix.

## 5. Conclusions

In this paper, the carbon fiber composite structure (6-layer laminate) manufactured using the 3D printing technology (FDM method) was presented. The small thickness of the carbon fibre bundles resulted in the heterogeneity of the sample structure. The analyses were focused on the determination of the influence of the elevated temperature on the mechanical characteristics of the additive manufactured sample. Both experimental and numerical analyses were performed at a stable humidity level, that was ensured by the chamber.

The mechanical behaviour of 3D printed CFRP under the global elevated temperature inside the chamber was modelled using the Abaqus software. The accuracy of the numerical model has been validated by means of convergence analysis and mesh resolution (grid independent). The convergence has been achieved by setting seeds size at a similar distance as 0.25 on every edge. The result from numerical data was compared with the experimental one and a good correlation was obtained. The mean difference between numerical and experimental values is about 18% (the average strain difference equal to 5.7 × 10${}^{-6}$ m/m) and it was probably due to the heterogeneity of the sample structure. The increasing strain differences for temperatures higher than 38 ${}^{{}^{\circ}}$C can be also related to the processes that occur in the matrix material and the weakness of bondings between fibres and matrix in the composite structure. It was confirmed by the fracture process observed for samples (with the same arrangement of layers) on which the tensile test was performed.

The heat deflection temperature (HDT) of CFRP from the investigation is found at approximately 42 ${}^{{}^{\circ}}$C while the DMA investigation of pure PLA used as matrix agent in CFRP has Tg at around 65 ${}^{{}^{\circ}}$C. This Tg is obtained from the curves in the glass transition region which are marked by an area around significant descent of storage modulus (65 ${}^{{}^{\circ}}$C) with an abrupt increased tan δ and loss modulus.

The lower HDT and Tg values of CFRP compared to neat PLA are due to the lower storage (elastic) modulus in the glassy region. As temperature increases, long-ranged coordinated molecular motion (α-relaxation) is activated and the onset of the glass transition triggers the refolding of polymer chains because their mobility increases. This increasing entropy is the main reason for shrinking. Furthermore, additional filler put off PLA chains due to lack of interaction between them. Moreover, extra reinforcement also acts as a weak point in the material which leads to the formation of shorter chains of PLA. The contraction of the CFRP sample at temperatures above the deflection temperature is a result of enthalpic relaxation at the beginning of the glass transition in the storage modulus curve.

As temperature increases, the rise in the crystalline concentration of PLA does not always correspond to an increase in Tg of CFRP. Glass transition temperatures fall as crystalline concentration increases over a certain level.

The analyses presented in the paper are concerned with one chosen sample with a specific structure and geometric dimensions. However, the conclusions were supported by observations performed for the other CFRP samples manufactured under the same AM conditions. The differences between the samples were related to the carbon fibre volume fraction (from 15% to 25%) and geometrical properties (rectangular samples with different dimensions). For all samples the relationship between strain and temperature was similar and the characteristic strain curve shape was observed. For samples with the same carbon fibre volume fractions, the differences between strain values were equal to 6%, while for the samples with different carbon fibre volume fractions it increased to 16%.

## Figures and Tables

**Table 1:** Material Properties of the fiber and matrix.

	Volume Fraction [%]	Young’s Modulus [Pa]	Shear’s Modulus [Pa]	Poisson’s Ratio [-]	Density [kg/m^3^]
Fiber	15.05	230 × 10^9^	17 × 10^9^	0.2	1760
Matrix	84.95	2.315 × 10^9^	24 × 10^8^	0.3	1240

**Table 2:** Elastic Properties of the CFRP composite.

E_11_ [Pa]	E_22_ [Pa]	E_33_ [Pa]	*ν*_12_ [-]	*ν*_13_ [-]	*ν*_23_ [-]	G_12_ [Pa]	G_13_ [Pa]	G_23_ [Pa]
36,580	2720	2720	0.3	0.3	0.3	14,070	14,070	14,070

**Table 3:** Thermal properties of the CFRP composite.

*α*_11_ [m/mK]	*α*_22_ [m/mK]	*κ*_11_ [m/mK]	*κ*_22_ [m/mK]	*C_v_* [J/kgK]
3.09 × 10${}^{6}$	7.41 × 10${}^{5}$	1.62	0.15	1438.91

**Table 4:** Polynomial constants (×10${}^{-3}$).

C${}_{1}$	C${}_{2}$	C${}_{3}$	C${}_{4}$
−0.000002011647241	0.000108722592509	0.001514056918440	−0.172701442544788

## Data Availability

Not applicable.
